# Regulation of the miR-19b-mediated SOCS6-JAK2/STAT3 pathway by lncRNA MEG3 is involved in high glucose-induced apoptosis in hRMECs

**DOI:** 10.1042/BSR20194370

**Published:** 2020-06-30

**Authors:** Fan Xiao, Lan Li, Jing-Song Fu, Yu-Xiang Hu, Rong Luo

**Affiliations:** Department of Ophthalmology, Jiangxi Provincial People’s Hospital Affiliated to Nanchang University, Nanchang 330006, Jiangxi Province, China

**Keywords:** DR, hRMECs, MEG3, miR-19b, SOCS6

## Abstract

**Objective:** Diabetic retinopathy (DR) is one of the most severe and common complications of diabetes mellitus. The present study aimed to investigate the molecular mechanism of MEG3, miR-19b and SOCS6 in human retinal microvascular endothelial cells (hRMECs) under high glucose conditions. **Methods:** HRMECs were cultured in 5 or 30 mM D-glucose medium. qRT-PCR and Western blotting were used to determine the mRNA expression and protein levels. MTT assay and flow cytometry analysis were performed to detect the viability and apoptosis of hRMECs, respectively. TNF-α, IL-6 and IL-1β levels in cell supernatants were detected by ELISA. The activity of caspase-3/7 was also determined. A luciferase reporter assay was performed to confirm the targeting relationship between miR-19b and SOCS6, as well as MEG3 and miR-19b. **Results:** Our study demonstrated that miR-19b was increased and SOCS6 was decreased in HG-induced hRMECs. Knockdown of SOCS6 inhibited cell viability and reversed the promotion of cell viability induced by knockdown of miR-19b. Additionally, miR-19b directly targeted and negatively regulated SOCS6. Moreover, miR-19b promoted the cell apoptosis rate and caspase-3/7 activity and increased inflammatory factors through the SOCS6-mediated JAK2/STAT3 signalling pathway. In addition, MEG3 attenuated HG-induced apoptosis of hRMECs by targeting the miR-19b/SOCS6 axis. **Conclusion:** These findings indicate that MEG3 inhibited HG-induced apoptosis and inflammation by regulating the miR-19b/SOCS6 axis through the JAK2/STAT3 signalling pathway in hRMECs. Thus, these findings might provide a new target for the treatment of DR.

## Introduction

As one of the most severe and common complications of diabetes mellitus (DM), diabetic retinopathy (DR) may result in retinal injury in diabetic patients caused by hyperglycaemia [1]. The high glucose environment damages the neurons and small vessels of the retina; as a result, the protective function of capillaries is lost, which remains a leading cause of various fundus lesions, even visual loss [2]. Increasing numbers of novel methods and drugs have been applied to the treatment of DR, and intravitreal administration of vascular endothelial growth factor inhibitors (anti-VEGFs) is currently the main therapeutic method for the early and advanced stages of DR. In addition, laser surgery, vitrectomy and steroid treatment are also conducted to manage microvascular complications [3]. Human retinal microvascular endothelial cells (hRMECs) are located in the lumen of retinal blood vessels and are closely related to various retinal vascular diseases, such as retinopathy of prematurity, diabetic macular oedema and proliferative retinopathy [4]. However, the underlying mechanism of hRMECs in DR remains unclear.

MicroRNAs (miRNAs) are small non-coding RNAs that regulate gene expression by binding target mRNAs [5]. Recently, miRNAs have been proven to be associated with DR and other microvascular diabetic complications, and genetic variations in miRNA-related genes have been confirmed [6]. It was reported that miR-19b was up-regulated in kidney exosomes and enriched in a chronic model. Moreover, overexpressed miR-19b in the urine was detected in patients with diabetic nephropathy, and it was also related to the severity of tubulointerstitial inflammation [5]. However, limited research has focused on miR-19b in DR, and its mechanism remains unclear.

Long non-coding RNAs (lncRNAs) are identified as a group of RNA transcripts containing more than 200 nucleotides that are not able to be translated into protein products [7]. It has been demonstrated that lncRNAs play important roles as competing endogenous RNAs (ceRNAs) in regulating gene expression. Additionally, increasing evidence indicates that, through miRNA response elements, miRNAs can interact with lncRNAs via the ceRNA network [8]. Previous studies suggested that lncRNAs contribute to the most important physiological processes in humans, plants and animals [9]. In addition, recent genetic evidence has also demonstrated that lncRNAs play regulatory roles in various diseases, such as cardiovascular disease, tumours and DR [10]. Although many lncRNAs have been identified in DR, very few lncRNAs have been confirmed to participate in the regulation and pathogenesis of DR. For instance, human retinal endothelial cells (HRECs) induced by high glucose could be inhibited by lncRNA SNHG7 [11], high glucose-induced apoptosis of the human retinal pigment epithelial (RPE) cell axis could be regulated via lncRNA IGF2AS [12], and lncRNA BANCR was overexpressed in patients with DR and promoted the pathogenesis of RPE cells [13]. LncRNA maternally expressed gene 3 (MEG3) is considered an important human gene and is located on chromosome 14q32.3 [10]. According to the finding by Qiu et al., the level of lncRNA MEG3 was dramatically down-regulated in retinas of STZ-induced diabetic mice, and high glucose induced oxidative stress in endothelial cells [14]. The latest research also pointed out that lncRNA MEG3 was significantly reduced in DR and contributed to the pathogenesis of this disease; that is, it may act as a prospective target for DR [9].

Based on the information above, we performed a study to investigate the molecular mechanism of MEG3 and miR-19b and their actions in DR. In our study, we demonstrated for the first time that MEG3 suppressed high glucose-induced apoptosis of hRMECs by sponging miR-19b through regulation of SOCS6/JAK2/STAT3 signalling. The results might imply the importance of MEG3 and miR-19b in the pathogenesis and development of DR and provide new promising therapeutic strategies for DR.

## Materials and methods

### Cell culture and treatment

Human retina microvascular endothelial cells (hRMECs) were purchased from Cell Biologics (Chicago, IL, U.S.A.) and were maintained in endothelial cell medium (ScienCell, San Diego, CA, U.S.A.) containing 10% foetal bovine serum (FBS), 100 μg/ml streptomycin and 100 U/ml penicillin (Invitrogen, Carlsbad, CA). All cells were placed at 37°C with 5% CO_2_ in an appropriate humidity, and the medium was renewed two or three times a week.

HRMECs were divided into a high glucose (HG) group and a normal glucose (NG) group. The HG group was subjected to medium with 30 mM D-glucose, while the normal group was treated with 5 mM D-glucose. Both groups were incubated for 48 h (5% CO_2_) at 37°C.

### Cell transfection

After different treatments, both groups were transfected with miR-19b mimic, inhibitor, pcDNA3.1-SOCS6 (p-SOCS6), pcDNA3.1-MEG3 (p-MEG3) and corresponding negative controls (NC, Thermo Fisher Scientific, Inc.) for 48 h using Lipofectamine 3000 reagent (Invitrogen, Carlsbad, CA, U.S.A.). The sequences were as follows: miR-19b inhibitor, sense 5′-UUC UCC GAA CGU GUC ACG UTT-3′, anti-sense 5′-UGA CAC GUU CGG AGA ATT-3′; miR-19b mimic, sense 5′-AGU UUU GCA UGG AUU UGC AC-3′ and anti-sense 5′-UUU GCA UGG AUU UGC ACA UU-3′.

### Methyl thiazolyl tetrazolium (MTT) assay

The transfected cells were cultured on 96-well plates at a density of 2 × 10^4^ cells/well, 10 μl MTT solution was added, and then incubated for 48 h at 37°C. Briefly, the medium was discarded and replaced with 150 μl DMSO. Finally, an ELX800 UV universal microplate reader (Olympus Optical Co., Ltd., Tokyo, Japan) was used to detect the absorbance of each well at 490 nm.

### Flow cytometry analysis of cell apoptosis

After D-glucose treatment for 48 h, hRMECs were harvested and washed with phosphate-buffered saline (PBS, Gibco). Cells were cultured with Annexin V-FITC for 20 min in the dark at room temperature, and propidium iodide (PI) was added. Afterwards, early apoptosis (Annexin V positive) and late apoptosis (Annexin V + PI positive) cells were quantified by FACSCalibur (Becton-Dickinson, Mountain View, CA, U.S.A.).

### Caspase-3/-7 activation

The activity of caspase-3/-7 was measured by a Caspase-Glo 3/7 assay kit (Promega, U.S.A.) according to the manufacturer’s instructions as reported elsewhere [15].

### RNA extraction and quantitative real-time PCR (qRT-PCR)

Briefly, hRMECs were collected, and TRIzol reagent (Invitrogen; Thermo Fisher Scientific, Inc.) was used to extract total RNA. For extraction of mRNA, the PrimeScript RT reagent Kit (TAKARA) was used for reverse transcription, and relative mRNA expression levels were calculated by the One Step SYBR PrimeScriptPLUS RT-RNA PCR Kit (TaKaRa Biotechnology, Dalian, China). MiRNAs were reverse-transcribed using the All-in-One-miRNA First-Strand cDNA Synthesis Kit (QP015, GeneCopoeia, U.S.A.) and detected by the miScript SYBR Green PCR Kit (Qiagen, Valencia, CA, U.S.A.) with the Stratagene Mx3000P real-time PCR system (Stratagene, La Jolla, California, U.S.A.). The following primers were used in PCRs: F 5′-GCATTAAGCCCTGACCTTTG-3′ and R 5′-TCCAGTTTGCTAGCAGGTGA-3′ for MEG3; F 5′-CGGAATTCATGAAGAAAATCAGTCTGAA-3′ and R 5′-CGGAATTCTCAGTAGTGCTTCTCCTGCA-3′ for SOCS6; 5′-F GGGTGTTCATCCATTCTC-3′ and R 5′-CCCAGCATCTTGTGTTTC-3′ for TNF-α; F 5′-CAAAGCCAGAGTCCTTCAG-3′ and R 5′-GATGGTCTTGGTCCTTAGC-3′ for IL-6; F GCAGGCAGTATCACTCATTG and R 5′-CACACCAGCAGGTTATCATC-3′ for IL-1β; F 5′-ACAGAGCCTCGCCTTTGC-3′ and R 5′-GAGGCGTACAGGGATAGCAC-3′ for β-actin; and F 5′-TGCGGGTGCTCGCTTCGGCAGC-3′ and R 5′-CCAGTGCAGGGTCCGAGGT-3′ for U6. The relative expression levels of miRNAs or mRNAs were calculated by the 2^−ΔΔCq^ method, and all gene expression levels were normalized to the level of the internal control β-actin or U6 for mRNA or miRNA, respectively.

### Western blot analysis

HRMECs were collected and extracted by RIPA lysis buffer (89901, Thermo Scientific, U.S.A.) with protease inhibitor cocktail (Merck KGaA, Darmstadt, Germany). A BCA™ Protein Assay Kit (Pierce, Appleton, WI, U.S.A.) was used to evaluate the protein levels. Equal amounts of proteins were loaded on 10% SDS-PAGE gels (Thermo Scientific), transferred to polyvinylidene fluoride (PVDF) membranes (Millipore, Bedford, Massachusetts, U.S.A.) and blocked with 5% non-fat milk at room temperature for 1 h. Afterwards, membranes were subjected to immunoblotting with primary antibodies (all purchased from Abcam, Cambridge, MA, U.S.A.) against SOCS6 (ab197335, 1/500), p-JAK2 (ab195055, 1/500) JAK2 (ab39636, 1/1000), p-STAT3 (ab32143, 1/1000) STAT3 (ab119352, 1/5000), PCNA (ab92552, 1/1000), Cyclin D1 (ab16663, 1/200), Cyclin E1 (ab33911, 1/1000), cleaved caspase-3 (ab32042, 1/500), Bcl-2 (ab59348, 1/500) and Bax (ab216494, 1/100) and incubated at 4°C overnight. Subsequently, the blots were washed with PBST, and the membranes were incubated with secondary peroxidase-linked goat anti-rabbit IgG H&L (1:5000, Santa Cruz, California, U.S.A.) at room temperature for 2 h. Signals were visualized by the ECL (Amersham Biosciences, Piscataway, NJ) method after washing, while optical densities of the bands were measured using ImageJ software (Bio-Rad).

### Enzyme-linked immunosorbent assay (ELISA)

The cell supernatant expression of TNF-α, IL-6 and IL-1β was determined by ELISA using commercially available kits strictly according to the manufacturer’s instructions.

### Luciferase reporter assay

Two types of 3′-UTRs (WT and MUT) of SOCS6 or MEG3 sequences containing the binding sequence of miR-19b were amplified and inserted into the p-MIR-report plasmid (Promega, Madison, WI). Then, hRMECs were co-transfected with the miR-19b vector (inhibitor or mimics) and SOCS6 luciferase reporter plasmid (WT-SOCS6 or MUT-SOCS6), MEG3 luciferase reporter plasmids (WT-MEG3 or MUT-MEG3) or NC (inhibitor or mimics) using Lipofectamine 3000 (GeneChem, Shanghai, China). After 48 h of cell transfection, the luciferase activity of each group was measured by a dual-luciferase reporter assay system (Promega, Madison, Wisconsin, U.S.A.). Luciferase activity of cells was normalized to Renilla luciferase activity.

### Statistical analysis

The data from three independent experiments are expressed as the mean ± SD. The difference between two groups was compared by Student’s *t*-test. Comparisons among three or more groups were conducted by using one-way analysis of variance (ANOVA) followed by Tukey’s post hoc test. *P*<0.05 was considered significant. All calculations were performed using SPSS 20.0 (IBM, Chicago, IL, U.S.A.).

## Results

### Up-regulated miR-19b and down-regulated SOCS6 in hRMECs under high glucose conditions

**Figure 1:**
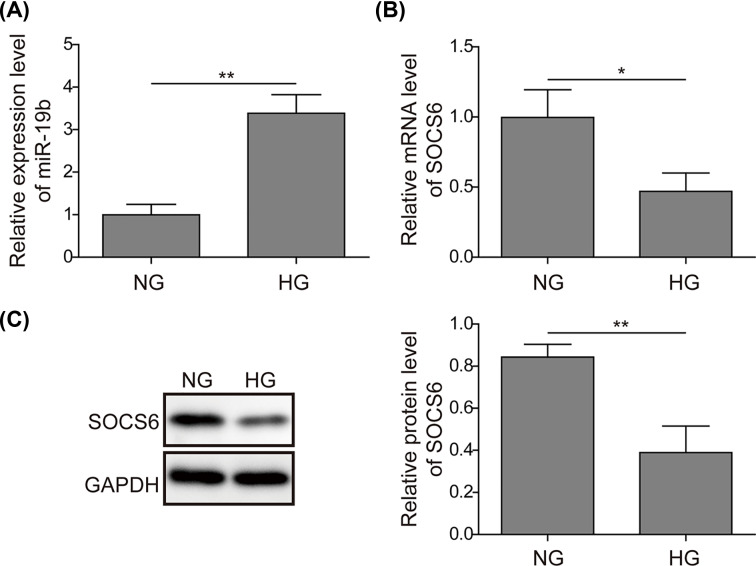
Up-regulated miR-19b and down-regulated SOCS6 in hRMECs under high glucose conditions (**A**) Expression of miR-19b was detected by qRT-PCR in the high glucose (HG) group and normal glucose (NG) group. (**B**) The mRNA expression level of SOCS6 was determined by qRT-PCR in the HG and NG groups. (**C**) Relative protein levels of SOCS6 were calculated by Western blotting in the HG and NG groups; *n*=3; *P<0.05, **P<0.01.

First, the expression levels of miR-19b and SOCS6 were measured under high glucose (HG) and normal glucose (NG) conditions by qRT-PCR and Western blotting, respectively. As shown in Figure 1A, miR-19b was highly up-regulated under HG compared with NG. However, the qRT-PCR and Western blotting results suggested that the mRNA and protein levels of SOCS6 were obviously decreased under HG (Figure 1B,C). These results suggest that miR-19b and SOCS6 are dysregulated, indicative of their regulatory roles in HG-induced hRMECs.

### MiR-19b promotes high glucose-induced apoptosis and inflammation of hRMECs

**Figure 2:**
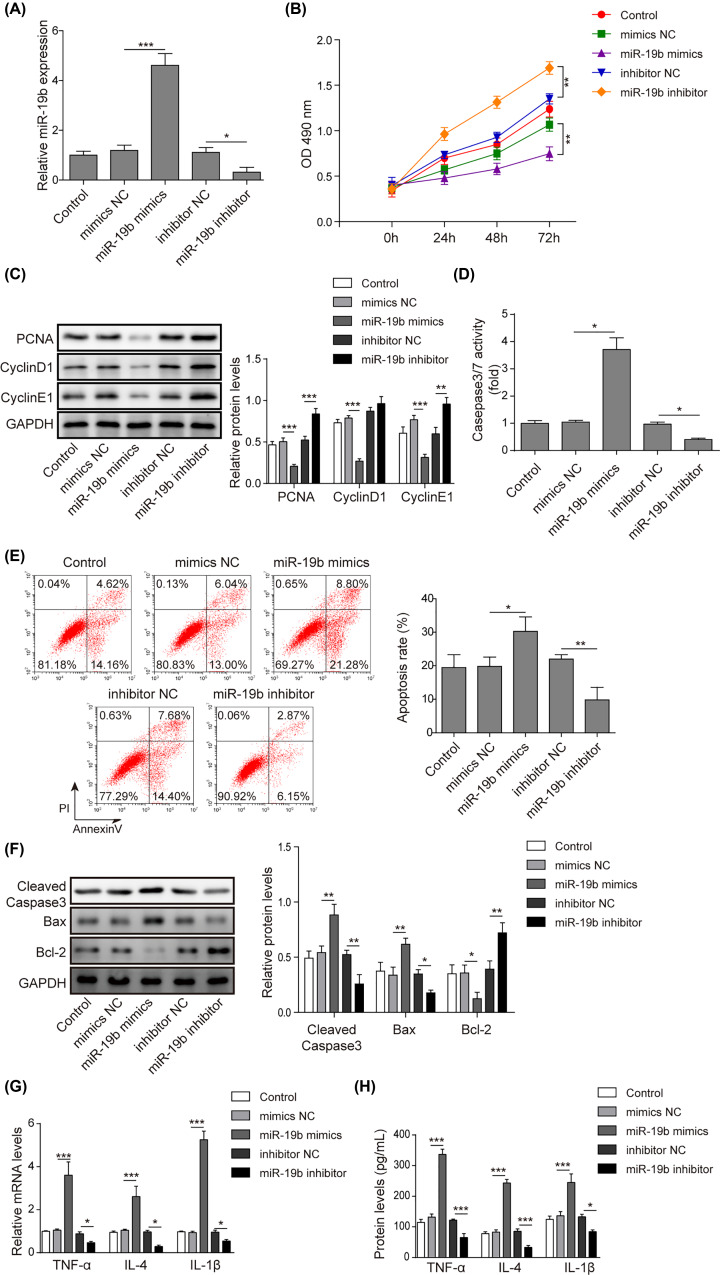
MiR-19b promotes HG-induced apoptosis and inflammation of hRMECs HRMECs were transfected with miR-19b mimics, miR-19b inhibitor and NCs. (**A**) The transfection efficiency was examined by qRT-PCR assay, which revealed that miR-19b mimics up-regulated miR-19b and miR-19b inhibitor down-regulated miR-19b successfully. (**B**) MTT assay was conducted to evaluate the viability of HG-treated hRMECs. (**C**) The protein levels of the proliferation-related molecules CNA, Cyclin D1 and Cyclin E1 were determined by Western blotting assay in HG-induced hRMECs. (**D**) The activity of caspase-3/7 was measured by commercial kits. (**E**) Flow cytometry analysis was applied to detect cell apoptosis in HG-induced hRMECs. (**F**) Western blotting assays were conducted to measure the protein levels of the apoptosis-related cleaved caspase-3, Bax and Bcl-2 in HG-induced hRMECs. (**G and H**) The secretion of inflammatory factors (TNF-α, IL-6 and IL-1β) in HG-induced hRMECs was measured by qRT-PCR and ELISA; *n*=3, *P<0.05, **P<0.01, ***P<0.001.

To explore the effects of miR-19b on the apoptosis of hRMECs under high glucose conditions, we overexpressed and knocked down miR-19b by transfection with miR-19b mimics and miR-19b inhibitor, respectively. As shown in Figure 2A, the expression of miR-19b in hRMECs was significantly enhanced by transfection with miR-19b mimics and was remarkably suppressed by the miR-19b inhibitor compared to the corresponding NC groups. Subsequently, the results on cell viability detected by the MTT assay showed that overexpression of miR-19b inhibited cell viability, while knockdown of miR-19b promoted cell viability (Figure 2B). The protein levels of the proliferation-related molecules CNA, Cyclin D1 and Cyclin E1 were tested, and the results showed that overexpression of miR-19b remarkably decreased the above protein levels, while inhibition of miR-19b significantly increased them (Figure 2C). Moreover, the activity of caspase-3/7 was markedly elevated by transfection with miR-19b mimics but was obviously decreased by inhibition of miR-19b (Figure 2D). In addition, the cell apoptosis rate was markedly promoted by the overexpression of miR-19b and inhibited by the knockdown of miR-19b (Figure 2E). To further confirm this result, apoptosis-related proteins, including cleaved caspase-3, Bax and Bcl-2 were examined by Western blotting (Figure 2F). The expression of cleaved caspase-3 and Bax was dramatically increased, and the expression of Bcl-2 was greatly reduced in the miR-19b mimics group, while miR-19b inhibition demonstrated the opposite effects. In addition, both the cell protein levels and mRNA expression of TNF-α, IL-6 and IL-1β were remarkably increased by miR-19b overexpression, and the opposite results were observed in the miR-19b inhibitor group (Figure 2G,H). These results indicated that miR-19b suppressed cell proliferation and aggravated apoptosis and the inflammatory response in HG-induced hRMECs.

### miR-19b negatively regulates SOCS6

**Figure 3:**
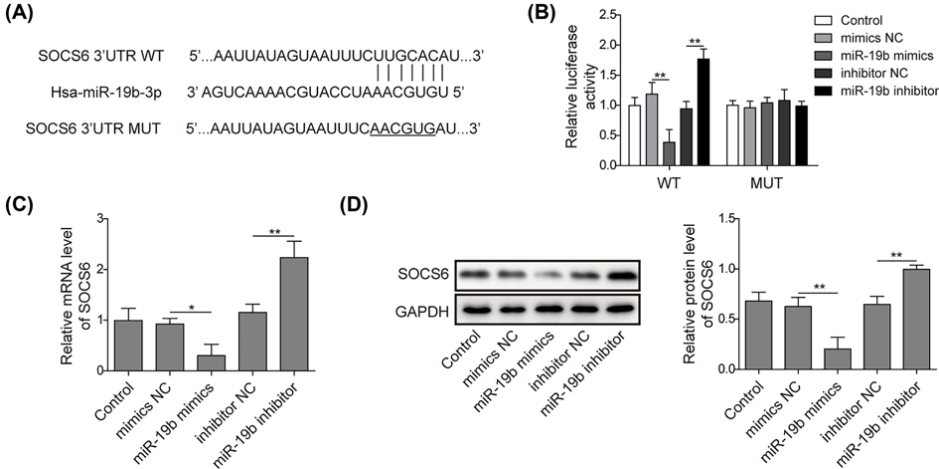
MiR-19b negatively regulates SOCS6 (**A**) The predicted binding sites between miR-19b and SOCS6 are illustrated. (**B**) The targeting relationship between miR-19b and SOCS6 was identified by luciferase reporter assay. (**C**) HRMECs were divided into five groups and transfected with miR-19b mimics, miR-19b inhibitor or NCs. The mRNA expression of SOCS6 was detected by qRT-PCR. (**D**) Protein levels of SOCS6 were measured by Western blotting; *n*=3, *P<0.05, **P<0.01.

Next, we explored the mechanism by which miR-19b promoted the apoptosis of hRMECs. According to bioinformatics analysis, miR-19b could directly bind to the SOCS6 (WT) 3′-UTR (Figure 3A). According to the results of the luciferase reporter assay, the transfection of miR-19b mimics significantly reduced the fluorescence intensity of WT-SOCS6, which was increased by the miR-19b inhibitor. However, the fluorescence intensity of MUT-SOCS6 was barely affected by either of them (Figure 3B). Furthermore, hRMECs were transfected with the miR-19b inhibitor or miR-19b mimics, and the expression of SOCS6 was detected. The results showed that overexpression of miR-19b markedly suppressed the expression of SOCS6, while its knockdown considerably up-regulated the SOCS6 level (Figure 3C,D). Thus, miR-19b targeted and negatively regulated SOCS6.

### miR-19b regulates apoptosis and inflammation by targeting SOCS6 through the JAK2/STAT3 signalling pathway in HG-induced hRMECs

**Figure 4:**
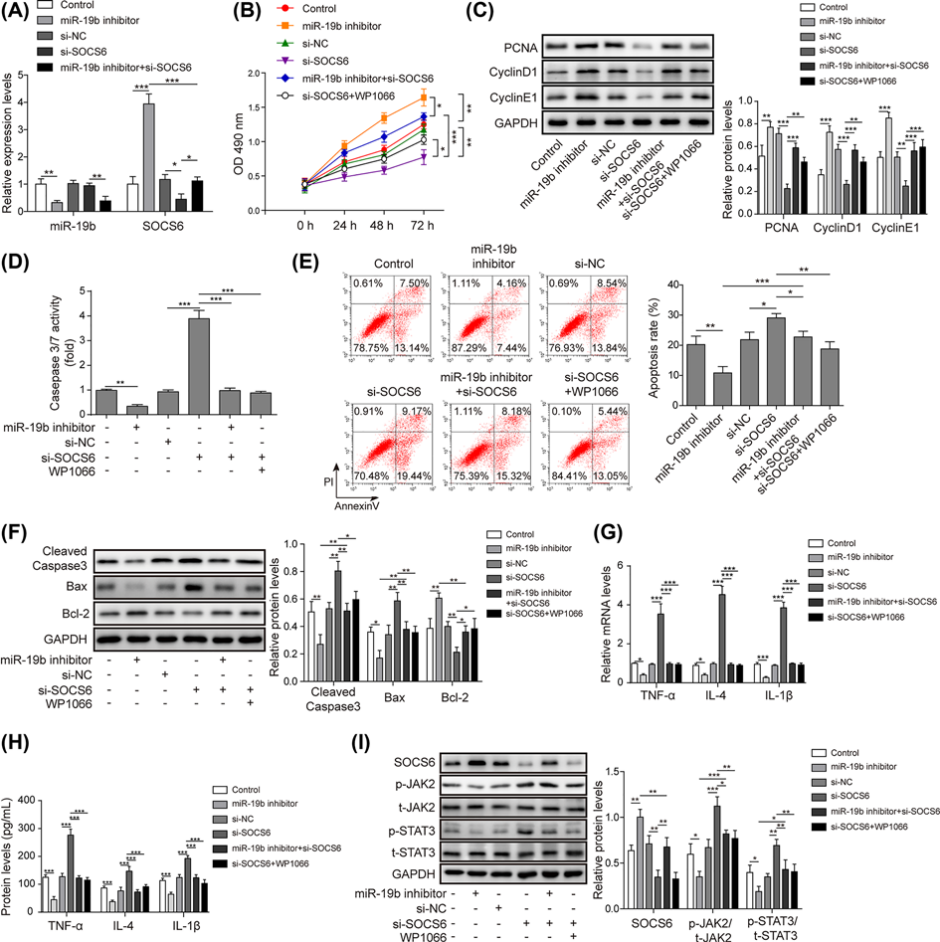
MiR-19b regulates apoptosis and inflammation by targeting SOCS6 via the JAK2/STAT3 signalling pathway in HG-induced hRMECs (**A**) HRMECs were transfected with si-SOCS6 and/or miR-19b inhibitor. Transfection efficiency was detected by qRT-PCR. (**B**) HRMECs were transfected with miR-19b inhibitor, si-SOCS6, miR-19b inhibitor and si-SOCS6, si-SOCS6 and treated with WP1066 or si-NC. Cell viability was tested by the MTT assay. (**C**) The protein levels of the proliferation-related molecules CNA, Cyclin D1 and Cyclin E1 in HG-induced hRMECs were determined by Western blotting assay. (**D**) The activity of caspase-3/7 in HG-induced hRMECs was measured by commercial kits. (**E**) Cell apoptosis in HG-treated hRMECs was evaluated by flow cytometry analysis. (**F**) The levels of the apoptosis-related proteins cleaved caspase-3, Bcl-2 and Bax in HG-induced hRMECs were measured by Western blotting. (**G** and **H**) The secretion of inflammatory factors (TNF-α, IL-6 and IL-1β) in HG-induced hRMECs was measured by qRT-PCR and ELISA. (**I**) The protein levels of the signalling pathway components SOCS6, phosphorylated JAK2 (p-JAK2), total JAK2 (t-JAK2), phosphorylated STAT3 (p-STAT3) and total STAT3 (t-STAT3) were detected by Western blotting; *n*=3, *P<0.05, **P<0.01, ***P<0.001.

To explore whether JAK2/STAT3 was involved in the effect of the miR-19b/SOCS6 axis on HG-induced hRMECs and identify its role in the whole process, SOCS6 was knocked down by transfection with si-SOCS6, and the JAK2/STAT3 signalling pathway was inhibited by treatment with WP1066. As shown in Figure 4A, si-SOCS6 reversed the promotion of SOCS6 expression induced by the miR-19b inhibitor, but the expression of miR-19b was not affected by si-SOCS6. Interestingly, the knockdown of SOCS6 reversed the promoting effects of miR-19b knockdown on the viability of HG-induced hRMECs (Figure 4B). Similarly, the protein levels of the proliferation-related molecules CNA, Cyclin D1 and Cyclin E1 were all stimulated by miR-19b inhibition, which was reversed by inhibition of SOCS6 (Figure 4C). Moreover, treatment with WP1066 rescued the protein levels of CNA, Cyclin D1 and Cyclin E1, which were suppressed by inhibition of SOCS6. Furthermore, the activity of caspase-3/7 and cell apoptosis were tested, and the results revealed that the knockdown of SOCS6 enhanced cell apoptosis and reversed the anti-apoptotic effect of the miR-19b inhibitor on HG-induced hRMECs (Figure 4D,E). Additionally, detection of the expression of apoptosis-related proteins by Western blotting assay showed a similar change trend (Figure 4F). Moreover, the secretion of inflammatory factors (TNF-α, IL-6 and IL-1β) was remarkably suppressed by inhibition of miR-19b, and si-SOCS6 reversed these effects (Figure 4G,H). In addition, transfection of the miR-19b inhibitor inhibited the JAK2/STAT3 signalling pathway, while si-SOCS6 played a positive role in this process and counteracted the suppressing effect of the miR-19b inhibitor on the JAK2/STAT3 signalling pathway. Nevertheless, WP1066, a JAK2 inhibitor, offset the effects of si-SOCS6 on the JAK2/STAT3 signalling pathway (Figure 4I). In addition, WP1066 reversed the anti-proliferative, pro-apoptotic and proinflammatory effects of si-SOCS6 in HG-treated hRMECs (Figure 4B–H). Therefore, miR-19b suppressed SOCS6 to modulate the proliferation, apoptosis and inflammation of HG-induced hRMECs through JAK2/STAT3 signalling.

### MEG3 attenuates HG-induced apoptosis and inflammation of hRMECs by sponging miR-19b

**Figure 5:**
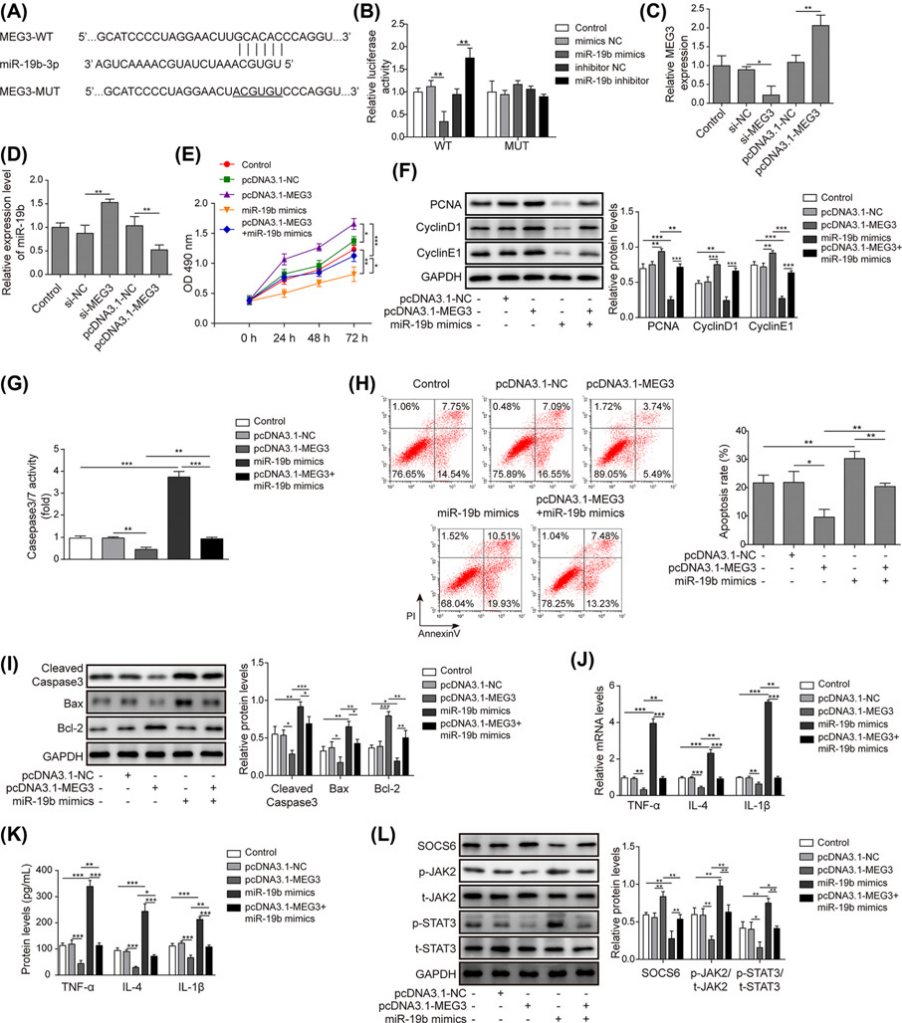
MEG3 attenuates HG-induced apoptosis and inflammation of hRMECs through miR-19b (**A**) The binding sites between miR-19b and MEG3 are shown. (**B**) The targeting relationship between miR-19b and MEG3 was identified by luciferase reporter assay in cells transfected with miR-19b mimics, miR-19b inhibitor or NCs. (**C**) The transfection efficiency of pcDNA3.1-MEG3 and si-MEG3 in hRMECs was validated by qRT-PCR. (**D**) Expression of miR-19b was determined by qRT-PCR in hRMECs transfected with pcDNA3.1-MEG3 and si-MEG3. (**E**) HRMECs were transfected with pcDNA3.1-MEG3, miR-19b mimics, pcDNA3.1-MEG3 and miR-19b mimics, or NC and then cultured in high glucose conditions. The viability of HG-induced hRMECs was evaluated by the MTT assay. (**F**) The protein levels of the proliferation-related molecules CNA, Cyclin D1 and Cyclin E1 in HG-induced hRMECs were determined by Western blotting assay. (**G**) The activity of caspase-3/7 in HG-induced hRMECs was measured by commercial kits. (**H**) The cell apoptosis rate in HG-induced hRMECs was evaluated by flow cytometry analysis. (**I**) The levels of the apoptosis-related proteins cleaved caspase-3, Bcl-2 and Bax in HG-induced hRMECs were measured by Western blotting. (**J** and **K**) The secretion of inflammatory factors (TNF-α, IL-6 and IL-1β) in HG-induced hRMECs was detected by qRT-PCR and ELISA. (**L**) The protein levels of the signalling pathway components SOCS6, p-JAK2, t-JAK2, p-STAT3 and t-STAT3 were detected by Western blotting; *n*=3, *P<0.05, **P<0.01, ***P<0.001.

Finally, the functional role of MEG3 in HG-induced hRMECs was investigated. Based on bioinformatics analysis, complementary binding sites between MEG3 and miR-19b were identified (Figure 5A). According to the luciferase reporter assay, the relative luciferase activity was markedly decreased in hRMECs transfected with miR-19b mimics, which was remarkably increased in hRMECs transfected with miR-19b inhibitor compared with the NCs in WT-MEG3 (Figure 5B). However, no significant difference was found in the MUT-MEG3 groups (Figure 5B). The qRT-PCR results revealed that the transfection of si-MEG3 and pcDNA3.1-MEG3 was successful (Figure 5C). Moreover, the expression of miR-19b was highly up-regulated by transfection with si-MEG3 but notably decreased with MEG3 overexpression (Figure 5D), suggesting that MEG3 targeted and negatively regulated miR-19b. Furthermore, according to the MTT results, MEG3 overexpression enhanced cell viability, but this effect was reversed by miR-19b overexpression (Figure 5E). In addition, the protein levels of the proliferation-related molecules CNA, Cyclin D1 and Cyclin E1 were all remarkably increased, but these levels were reversed by overexpression of miR-19b (Figure 5F). Moreover, overexpression of MEG3 inhibited the activity of caspase-3/7 and cell apoptosis, which was offset by overexpression of miR-19b (Figure 5G,H). Moreover, the overexpression of MEG3 also down-regulated the protein levels of Bax and cleaved caspase-3 and up-regulated the expression of Bcl-2, while these effects were counteracted by miR-19b mimics (Figure 5I). Additionally, both the cell supernatant levels and mRNA expression of TNF-α, IL-6 and IL-1β were remarkably decreased by overexpression of MEG3, and transfection of miR-19b mimics reversed this effect (Figure 5J,K). In addition, we also noted that the JAK2/STAT3 pathway was suppressed along with the up-regulation of SOCS6. As a result, the JAK2/STAT3 signalling pathway was inhibited, which rescued the promoting effect of miR-19b mimics on the JAK2/STAT3 signalling pathway (Figure 5L). Together, these results indicated that MEG3 regulated HG-induced apoptosis and inflammation through the miR-19b-mediated targeting of SOCS6 via the JAK2/STAT3 signalling pathway in hRMECs.

## Discussion

Diabetic retinopathy (DR) is a progressive disease that results in blindness, which imposes a substantial burden to society and reduces patient quality of life [16,17]. It is known that hRMECs play an important role in DR [18]. In recent decades, the roles of lncRNAs and miRNAs, as well as their interactions, in the development of DR have been reported in several studies. However, the mechanism of miR-19b, SOCS6 and lncRNA MEG3 in hRMECs apoptosis and the targeting relationship among them remain uncertain. In the present study, our data revealed that miR-19b aggravated HG-induced apoptosis, stimulated inflammatory factors and suppressed the proliferation of hRMECs by targeting the SOCS6-mediated JAK2/STAT3 signalling pathway. In addition, MEG3 attenuated the high glucose-induced apoptosis of hRMECs by sponging miR-19b.

Recently, it has been reported that the miR-19 family has diagnostic value in cancers by targeting tumour suppressive genes, such as prostate cancer, breast cancer, colorectal cancer and hepatocellular carcinoma [19]. Furthermore, miR-19b was proven to be involved in the regulation of cancer initiation, promotion and progression. In addition, it was revealed that miR-19b was a potential biomarker for diabetic cardiomyopathy [20]. In our study, miR-19b was up-regulated in HG-treated hRMECs, and miR-19b overexpression dramatically inhibited cell viability and enhanced cell apoptosis and inflammation. Similarly, according to Cai’s study, inhibition of miR-19b could promote the proliferation of hair follicle stem cells (HFSCs) [21]. All these studies supported our findings. However, different results have also been observed in other kinds of studies. For instance, miR-19b was shown to negatively regulate PLZF expression in goat mGSCs and induce goat mGSC proliferation [22]. Kong also pointed out that miR-19 promoted skeletal muscle cell differentiation cooperatively with miR-17 [23]. In addition, it was reported that miR-19b could suppress apoptosis and promote the proliferation of T-cell lines. As a result, miR-19b accelerated the cell cycle [24]. All these studies indicate that miR-19b may play different roles in cell apoptosis in different diseases. Therefore, further investigation is needed to confirm its role in DR.

Suppressor of cytokine signalling (SOCS) proteins have been proven to be intracellular inhibitors of the JAK/STAT signalling pathway [25]. As reported, up-regulation of SOCS3 inhibited MMP-9 expression in retinal microglia induced by high glucose [26]. According to Chen’s study, SOCS6 was identified as the target of miR-142-3p in primary human periodontal ligament cells [27]. In addition, SOCS6 was considered a negative regulator of cytokine receptor signalling. It was reported that SOCS6 could decrease the protein levels of p-STAT3 and HIF-1α in breast cancer and hepatocellular cancer [28]. We also found that miR-19b targeted and negatively regulated SOCS6 in DR, and Duncan’s study showed that SOCS protein expression blocked the JAK/STAT pathway and regulated cytokine signalling by inhibiting JAK activity [29]. Interestingly, the present research verified that silencing SOCS6 activated the JAK2/STAT3 signalling pathway. In addition, miR-19b suppressed SOCS6 to modulate proliferation, apoptosis and inflammation in HG-induced hRMECs through JAK2/STAT3 signalling, consistent with previous studies.

As a tumour suppressor, MEG3 has been found to suppress cancer development in various human cancer cells, including hepatocellular carcinoma, oesophageal cancer and breast cancer [30]. However, the role of MEG3 in DR is not clear. As stated in the report by Li et al. MEG3 enhanced cell proliferation and deactivated the miR-141-mediated AKT/mTOR signal pathway in LPS-treated chondrocytes [31]. Another report also found that the silencing of MEG3 could aggravate LPS-induced injury of lung cells [32]. Nevertheless, in the last decade, limited research has investigated the underlying molecular mechanism of MEG3 in diabetic retinopathy. In the present study, we first confirmed that MEG3 suppressed high glucose-induced cell apoptosis of hRMECs by negatively regulating miR-19b. Moreover, we further demonstrated that overexpression of MEG3 enhanced cell viability, reversing the inhibitory effect of miR-19b mimics. In summary, MEG3 decreased high glucose-induced apoptosis, the release of inflammatory factors and caspase-3/7 activity in hRMECs by repressing miR-19b.

## Conclusion

In conclusion, MEG3 targeted the miR-19b/SOCS6 axis to suppress high glucose-induced apoptosis, increase inflammatory factors and enhance caspase-3/7 activity in hRMECs through the JAK2/STAT3 signalling pathway. The present study demonstrated the molecular mechanism of MEG3, miR-19b and SOCS6 in the apoptosis and inflammation of HG-induced hRMECs, as well as their possible interactions. This finding might provide a new direction for the targeted therapy of DR.

## Abbreviations

ceRNAs: competing endogenous RNAs
DM: diabetes mellitus
DR: diabetic retinopathy
ELISA: Enzyme-linked immunosorbent assay
HG: high glucose
HRECs: human retinal endothelial cells
hRMECs: human retinal microvascular endothelial cells
lncRNA: long non-coding RNA
miRNAs: microRNAs
MEG3: maternally expressed gene 3
NG: normal glucose
qRT-PCR: quantitative real-time PCR
RPE: retinal pigment epithelial
SOCS: suppressor of cytokine signalling
